# Application of Logistic Regression and Artificial Intelligence in the Risk Prediction of Acute Aortic Dissection Rupture

**DOI:** 10.3390/jcm12010179

**Published:** 2022-12-26

**Authors:** Yanya Lin, Jianxiong Hu, Rongbin Xu, Shaocong Wu, Fei Ma, Hui Liu, Ying Xie, Xin Li

**Affiliations:** 1 Critical Care Medicine, Affiliated Hospital of Putian University, Putian 351100, China; 2 School of Mechanical, Electrical & Information Engineering, Putian University, Putian 351100, China; 3 Department of Emergency Medicine, Guangdong Provincial People’s Hospital, Guangdong Academy of Medical Sciences, Guangzhou 510080, China; 4 Department of Radiology, Guangdong Provincial People’s Hospital, Guangdong Academy of Medical Sciences, Guangzhou 510080, China

**Keywords:** artificial intelligence, machine learning, logistic regression, acute aortic dissection rupture

## Abstract

Logistic regression (LR) and artificial intelligence algorithms were used to analyze the risk factors for the early rupture of acute type A aortic dissection (ATAAD). Data from electronic medical records of 200 patients diagnosed with ATAAD from the Department of Emergency of Guangdong Provincial People’s Hospital from April 2012 to March 2017 were collected. Logistic regression and artificial intelligence algorithms were used to establish prediction models, and the prediction effects of four models were analyzed. According to the LR models, we elucidated independent risk factors for ATAAD rupture, which included age > 63 years (odds ratio (OR) = 1.69), female sex (OR = 1.77), ventilator assisted ventilation (OR = 3.05), AST > 80 U/L (OR = 1.59), no distortion of the inner membrane (OR = 1.57), the diameter of the aortic sinus > 41 mm (OR = 0.92), maximum aortic diameter > 48 mm (OR = 1.32), the ratio of false lumen area to true lumen area > 2.12 (OR = 1.94), lactates > 1.9 mmol/L (OR = 2.28), and white blood cell > 14.2 × 10^9^ /L (OR = 1.23). The highest sensitivity and accuracy were found with the convolutional neural network (CNN) model. Its sensitivity was 0.93, specificity was 0.90, and accuracy was 0.90. In this present study, we found that age, sex, select biomarkers, and select morphological parameters of the aorta are independent predictors for the rupture of ATAAD. In terms of predicting the risk of ATAAD, the performance of random forests and CNN is significantly better than LR, but the performance of the support vector machine (SVM) is worse than LR.

## 1. Introduction

Acute type A aortic dissection (ATAAD) refers to the vascular emergency when an intimal tear creates a false lumen in the ascending aorta [1]. This is an uncommon but life-threatening cardiovascular condition that allows for diagnosis, risk stratification, and management of aortic disease with the use of computed tomographic angiography (CTA) [2]. Harris et al. [3] found that the overall mortality rate for ATAAD was 5.8% at 48 h. For patients receiving conservative therapy, ATAAD had a mortality rate of 0.5% per hour (23.7% at 48 h). However, among those in the surgical group, 48-h mortality decreased to 4.4%. However, the lack of risk assessment of dissection rupture may affect medical decision-making and resource allocation for high-risk patients and even hinder their treatment.

It is vital to establish a simple risk prediction model that can quickly assess the risk of ATAAD rupture. In recent years, some scholars have devoted themselves to this research. Kuang et al. used multivariate analysis to study the predictive factors of preoperative mortality in patients with ATAAD and established a prediction model [4]. Wu et al. took the lead in using a machine learning algorithm (random forest) to predict the risk of patients with ATAAD and built a web page prediction model [5]. However, the previous studies generally have a small sample size, improper selection of variables, and differences in results.

Accumulating evidence has shown that logistic regression (LR) and artificial intelligence (AI) can generate disease prediction models with high prediction accuracy [6,7]. However, the traditional LR model is easy to underfit and the classification accuracy is moderate. The performance is low when the data feature is missing or the feature space is large [8]. Among artificial intelligence technologies, the support vector machine (SVM) has been the focus of machine learning in the field of aortic dissection, random forest (RF) shows its advantages in many fields, and the convolutional neural network (CNN) has been a research focus in recent years [9,10].

The present study aims to analyze the risk factors for ATAAD rupture based on the CTA imaging and clinical features using LR and AI algorithms to establish the ATAAD rupture prediction models.

## 2. Materials and Methods

### 2.1. Study Design, Patients

The present study was a retrospective case-control study. The protocol of the study was approved by the Ethics Committee of the Guangdong Provincial People’s Hospital, Guangdong Academy of Medical Sciences, China. Due to its retrospective design and anonymous characteristics, the requirement for informed patient consent was waived.

**Figure 1:**
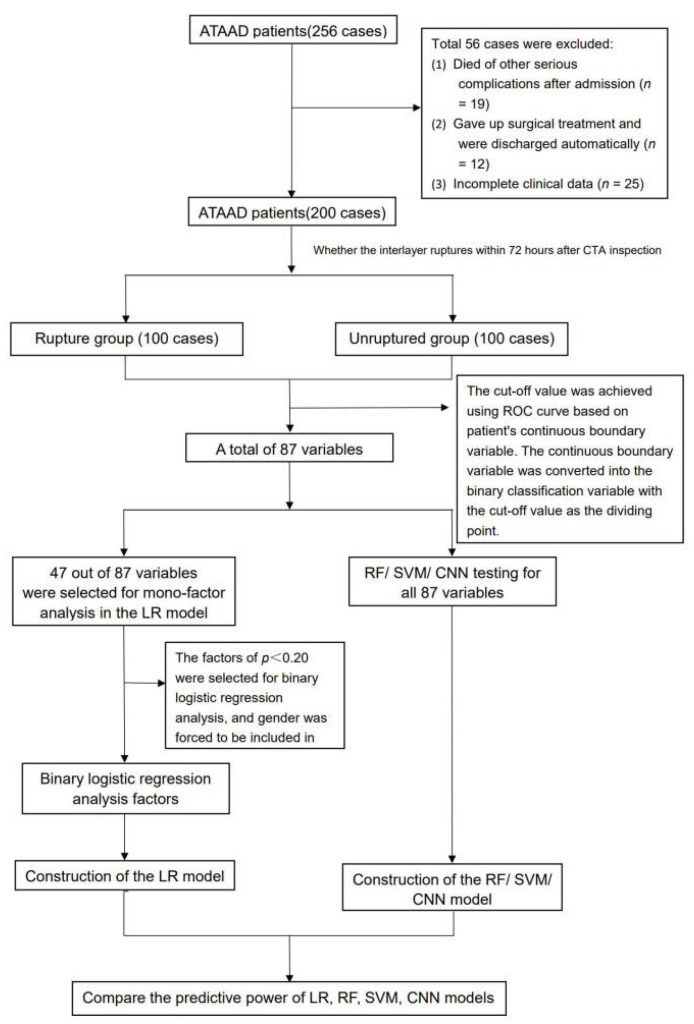
An overview of the flow through the study.

Patients diagnosed with ATAAD in the Department of Emergency of Guangdong Provincial People’s Hospital from 1 April 2012 to 31 March 2017 were screened. Inclusion criteria: (1) age ≥ 18 years, (2) onset time ≤ 14 days, (3) CTA diagnosed as ATAAD. Exclusion criteria: (1) age < 18 years; (2) ATAAD patients who died of other serious complications such as myocardial infarction or cerebral infarction caused by coronary artery dissection and branch dissection of aortic arch; (3) iatrogenic or traumatic aortic dissection; (4) patients with previous serious diseases of other systems, such as heart failure, uremia, liver cirrhosis, advanced malignant tumors, etc.; (5) patients with aortic dissection who refused active treatment. An overview of the flow through the study is given in Figure 1. Finally, 200 eligible patients were included in the final analysis.

### 2.2. Outcomes

The primary outcome was death due to the dissection rupturing within 72 h after the CTA. The rupture was diagnosed by the bedside color Doppler ultrasound examination [11], which showed a large amount of fluid in the pericardium, mediastinum, or chest and abdomen.

### 2.3. Data Collection

Patient data were collected from medical records, including demographic and patient measurements, such as patients’ clinical data, clinical symptoms, general conditions, complications, X-ray findings, color ultrasound results, laboratory tests, clinical results, etc.

### 2.4. Construction of the LR Model

The cut-off value was achieved using the receiver operating characteristic (ROC) curve. the factors with a *p* < 0.20 in univariate analysis were selected for multivariate analysis, and gender was forced to be included. The independent variable screening adopts the forward step method (Forward: LR) based on the likelihood ratio test.

### 2.5. Construction of the RF Model

The ATAAD rupture risk prediction model based on an RF algorithm was established with a training data set. We applied an RF algorithm for classification and regression trees (CART) as a meta-classifier to build an integrated classifier. The bootstrap sampling method was used to randomly extract *k* samples from the original training sample set *N* to generate new training subsets. The new training subsets were different from each other and then build *k* decision trees according to the *k* training subsets. For data whose response variable was categorical, the final classification of each record was determined by voting on the basis of the classification results of multiple trees [12].

Variable importance scores were used to evaluate the influence of variables on the occurrence of rupture. We sorted the variables according to their importance scores. Starting with the variable with the highest score, a stepwise RF analysis was performed, and the model was constructed.

### 2.6. Construction of the SVM Model

The goal of constructing an SVM model was to create an optimal classification boundary (highest-spaced hyperplane) in a high-dimensional space and distinguish different types of samples. The maximum interval hyperplane was the classification boundary, where the distance between the closest points reaches the maximum. Support vectors refer to the points in each class that are closest to the largest spaced hyperplane [12].

### 2.7. Construction of the CNN Model

Another alternative classifier in our study is CNN, which is a type of artificial neural network. Commonly used loss functions include root-mean-square error, negative log-likelihood, and cross-entropy. During the training of the network, the parameters $\left(W,b\right)$ were continuously corrected by the stochastic gradient descent method, and the error was propagated forward one by one. Then, the parameters $\left(W,b\right)$ of the network were updated layer by layer until the error was small enough or the loss function was optimal.

### 2.8. Statistical Analysis

The Shapiro-Wilk test was used to determine whether the data conformed to the normal distribution. Normal distribution data were expressed as (mean ± SD), and t-tests were used for comparison between groups. Non-normal distribution data were expressed as median (*M*) and interquartile range (*P*25, *P*75), and the difference was compared by two independent sample rank sum tests. Count data were expressed as the number of cases (percentage), and comparison between groups was analyzed using the *χ*^2^ test or Fisher exact probability. *p* < 0.05 was considered statistically significant. The LR model was performed by Statistical Package for Social Sciences (SPSS) 22.0 (IBM, Armonk, NY, USA). Python software was used to build RF, SVM, and CNN models. The performance measurement of the models was used to compare the generalization ability of the classifier. Precision and recall were more important than other evaluation indicators in disease risk prediction. We used the following indicators for the performance evaluation of the four models: AUC of ROC, accuracy, precision, specificity, recall, and F1-score.

## 3. Results

For the included patients, the average age was 53.30 ± 13.19 years and 160 of the subjects were males. Medical history included 155 cases of hypertension (77.50%), 55 cases of diabetes (27.50%), 132 cases of smoking (66.00%), and six cases of Marfan syndrome (3.00%). According to whether the interlayer ruptures occurred within 72 h after CTA inspection, the patients were divided into the rupture group (100 cases) and an unruptured group (100 cases). We summarized relevant clinical indicators as the risk factors related to dissection rupture, which are presented in Table 1.

### 3.1. LR Model

Univariate analysis was used to test the partial risk factors for rupture risk occurring within 72 h after CTA in the two groups of patients. Factors with *p* < 0.20 were selected for multiple logistic regression analysis, and finally, ten independent risk factors were included in the model (Table 2).

**Figure 2:**
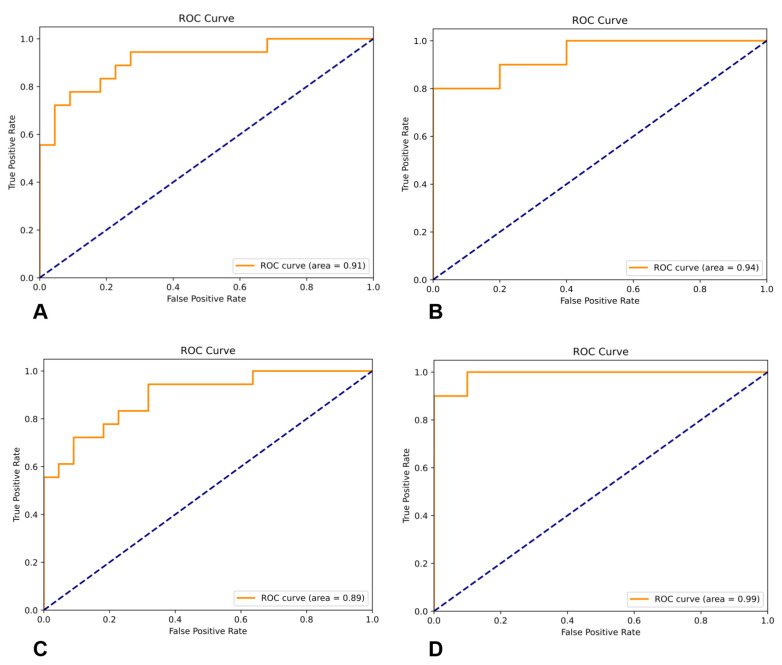
Receiver operating characteristic (ROC) curve of different models. (**A**) LR model variables. (**B**) RF model variables. (**C**) SVM model variables. (**D**) CNN model variables.

Based on the LR analysis results, an ATAAD rupture risk prediction formula is established: logit (*p*) = −5.82 + 1.69× (if age > 63 years) + 1.77× (if the patients were women) + 3.05 × (if having ventilator-assisted ventilation) + 1.59 × (if AST > 80 U/L) + 1.57 × (if no distortion of the inner membrane)+ 0.92 × (if aortic sinus diameter > 41 mm) + 1.32 × (if maximum diameter > 48 mm) + 1.94 × (ratio of false lumen area to true lumen area > 2.12) + 2.28 × (if Lac > 1.9 mmol/L) + 1.23 × (if WBC > 14.2 × 10^9^/L). The predicted rupture probability was a value derived from the LR equation. The observed rupture probability was the actual frequency of rupture observed in the case group. The prediction performance of the model was tested. The ROC AUC of the rupture risk score was 0.91 (95% CI: 0.87 to 0.95, *p* < 0.01), as shown in Figure 2A. The Hosmer–Lemeshow test *χ*^2^ = 3.38, *p* = 0.91, sensitivity was 0.83, accuracy was 0.85, precision was 0.90, F1-score was 0.88, specificity was 0.86, and recall was 0.90.

### 3.2. RF Model

**Figure 3:**
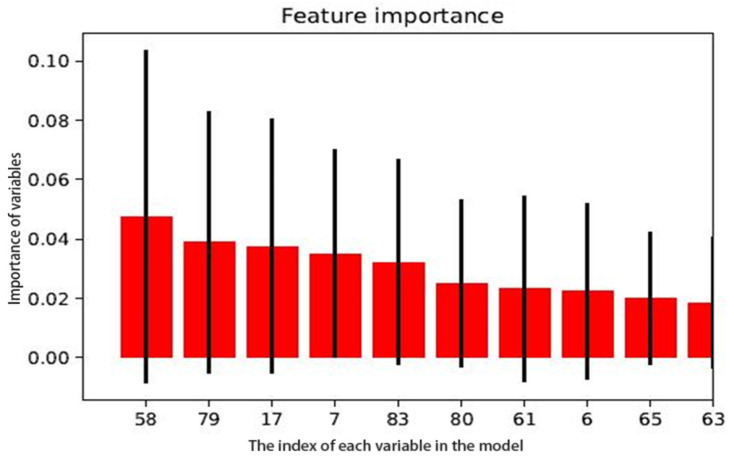
Importance scores of random forest model variables. ID58:PH, ID79: Lac > 1.9 mmol/L, ID17: false cavity area > 11.85 cm^2^, ID7: ventilator assisted ventilation, ID83: respiratory rate, ID80: maximum diameter > 48 mm, ID61: ratio of false cavity area to true cavity area > 2.12, ID6: FiO_2_, ID65: heart rate, ID63: cTnT.

The ATAAD rupture risk prediction model based on an RF algorithm was established with the training set. First, the sequence of the variables was ordered according to their importance (*p* value from low to high). Then, we performed a stepwise random forest analysis. The results showed that the error rate of the data outside the bag was the lowest when the number of variables was 10. Therefore, the top 10 variables of variable importance scores were included in the RF algorithm to establish the ATAAD rupture risk prediction model. The top 10 variables of importance score were: pH, lactates (Lac) > 1.9 mmol/L, false cavity area > 11.85 cm^2^, ventilator assisted ventilation, respiratory rate, maximum diameter > 48 mm, the ratio of false cavity area to true cavity area > 2.12, FiO_2_, heart rate, and cTnT (Figure 3).

The prediction performance of the model was tested. Its accuracy was 0.90, precision was 0.92, F1-score was 0.89, specificity was 0.91, recall was 0.95, and ROC AUC was 0.94 (Figure 2B).

### 3.3. SVM Model

The ROC AUC of the SVM model was 0.89, accuracy was 0.83, precision was 0.78, F1-score was 0.77, specificity was 0.85, and recall was 0.88 (Figure 2C).

### 3.4. CNN Model

The ROC AUC of the CNN model was 0.99, accuracy was 0.90, precision was 0.90, F1-score was 0.90, specificity was 0.90, and recall was 0.90 (Figure 2D). The performance comparison of the four models is shown in Table 3.

## 4. Discussion

ATAAD is characterized by acute onset, rapid progression, and high mortality. We all know that the earlier the surgery, the better the prognosis. However, there are always special circumstances, such as long distances and a sudden influx of patients. In addition, medical resources are unevenly distributed in developing countries and many sites are unable to perform aortic coarctation surgery on their own. Patients need to be referred to a superior hospital or to a superior hospital for expert assistance, which takes an average of 3 days [4]. Therefore, the inclusion criterion of this study was patients who were inoperable within 72 h after the CTA examination. So, in these situations, the doctors have to make a choice, which patient should be operated on first?

In the LR model, 10 clinical variables significantly predicted rupture risk in patients with ATAAD. Simple and highly discriminative scoring tools can be further generated to help physicians make better decisions and communicate better with patients. Given that aortic dissection involving the aortic valve or airway compression may lead to heart failure and further respiratory failure in patients, the risk weight of ventilator-assisted ventilation is highest. Such patients are critically ill with a high risk of rupture and surgery should be given priority.

In this study, the strong predictors identified by random forest included PH, Lac value > 1.9 mmol/L, false cavity area > 11.85 cm^2^, ventilator-assisted ventilation, etc. The decreased PH value and the increased Lac value are closely related to the ischemia, hypoxia, and shock of the body, suggesting the involvement of important branch arteries or rupture of the clamps, and such patients need priority surgical treatment. The importance score of ventilator-assisted ventilation in the random forest algorithm was also high, further confirming its importance in prediction.

Some morphological parameters of the aorta, including the maximum diameter of the aorta, the area or volume of the pseudo cavity, the ratio of the false cavity area to the true cavity area, and the state of the pseudo luminal thrombosis, are closely related to the prognosis of patients with acute aortic dissection [13,14,15]. Consistent with the above studies, we found that maximum diameter > 48 mm, the diameter of the aortic sinus > 41 mm, the area of false cavity > 11.85 cm^2^, and the ratio of false cavity area to true cavity area > 2.12 were important risk factors for the rupture of ATAAD. We speculate that the larger the diameter of the aorta, the larger the false lumen area, and the larger the ratio of the false lumen area to the vacuum lumen area, implying higher pressure and a thinner, and weaker aortic wall.

In addition to X-ray findings, we found that subjects of advanced age (>63 years old) were more likely to experience dissection rupture than younger ones, which is similar to other reported evidence [16,17]. In the present study, women with ATAAD were more likely to experience rupture within 72 h after CTA than men (adjusted OR = 1.77), which is not consistent with previous reports [18]. In the present study, the women’s average age is higher than men, which may be why the rupture rate among female patients was elevated.

Prior reports have shown that many biomarkers, including white blood cell count, platelet count, C-reactive protein, cardiac troponin T, N-terminal brain natriuretic peptide, D-dimer, fibrinogen, and matrix metalloproteinase, are closely related to the progress and prognosis of interlayer rupture [19,20,21]. Those biomarkers are associated with degeneration of the aortic vascular media. The wall of the aortic tube is then weakened, which finally results in dissection. In this study, the LR and RF algorithms also demonstrated that WBC > 14.2 × 10^9^/L and cTnT were important risk factors for ATAAD rupture.

As an emerging machine learning algorithm, the RF algorithm has a wide range of applications in disease risk assessment. For instance, Casanova et al. used the Jackson cardiac study cohort data, LR analysis, and RF algorithm to predict diabetes and found that the accuracy of the RF algorithm was higher than that of the LR analysis [17]. In the present study, the RF model had higher accuracy, precision, and F1-score than the LR model in predicting the probability of interlayer rupture risk, and its AUC was higher than the LR model. Therefore, the RF model had better overall performance compared with the LR model.

SVM is a machine learning method proposed by Vapnik et al. based on the principle of structural risk minimization in the mid-1990s [22]. Different from the LR algorithm, the SVM algorithm does not require a defined sample size. This method adopts the structural risk minimization criterion, which minimizes the sample point error and ensures structural risk minimization. It has the best classification and generalization effect [13]. The present study shows that the sensitivity of the SVM algorithm to predict interlayer fracture was higher than that of the LR analysis, but the accuracy was lower compared with the LR analysis.

CNN has a wide range of applications in medical image processing and data processing [15,23]. It has good fault tolerance, parallel processing, and self-learning capabilities, and runs faster than other deep-learning methods [24]. This study showed that CNN obtained the final classification result by predicting the data of a single variable according to the size of the comparison probability, and indirectly proved that the prediction performance of CNN for a single variable is more accurate. In this study, the performance of the CNN model is better than that of the LR model.

Medical decision support systems based on AI have received increasing attention from the public. However, some models have low explanatory power and are difficult to apply in clinical work. Therefore, medical experts must be involved in the entire process of data collection, modeling, and data analysis to ensure that the model is interpretable. The four models we explored have their advantages, which can help clinicians to improve the accuracy of early ATAAD rupture risk prediction. However, there are limitations to the study. As a retrospective study, we cannot avoid the information bias of collecting data from medical records. Next, the sample size is small. A prospective, larger-scale study is needed to confirm our findings.

## 5. Conclusions

In the present study, we found that age, sex, select biomarkers, and select morphological parameters of the aorta are independent predictors for the rupture of ATAAD within 72 h after CTA. In terms of predicting the risk of ATAAD, the performance of RF and CNN is significantly better than LR, but the performance of SVM is worse than LR.

## Figures and Tables

**Table 1:** Risk factors for acute type A aortic dissection.

Risk Factors			
Age	Ischemic manifestation of superior mesenteric artery	Presence of severe aortic regurgitation	Creatine kinase isoenzyme value
Women	Aortic sinus diameter	Presence of a large amount of pericardial effusion presence	Age > 63 years ^b^
EF	Sinus canal junction diameter	High blood pressure presence	Aortic sinus diameter > 41 mm
PH	Widest diameter ^d^	Diabetes	Sinus canal junction diameter > 38 mm
Lac	Arc length of false cavity ^d^	Smoking history	Arc length of false cavity > 119 mm ^d^
PaO_2_	Radian of false cavity ^d^	Marfan syndrome	Radian of false cavity > 4.42 rad ^d^
PaCO_2_	False cavity area ^d^	Heart rate	Length of aortic dissection > 534 mm
FiO_2_	Ratio of false lumen area to true lumen area ^d^	Respiratory rate	False cavity area > 11.85 cm^2 d^
WBC	Maximum breaking diameter	Shock	Ratio of false lumen area to true lumen area > 2.12 ^d^
NEUT	Length of aortic dissection	Ventilator assisted ventilation	Initial break diameter > 15.5 mm
PLT	Full-length aorta	Chest pain	Number of branch vessels involved > 3
cTnT	Ratio of aortic dissection length to aortic length	Syncope	Maximum diameter > 48 mmd
NT-proBNP	No thrombus in the false cavity	Mental symptoms presence	Time of onset to the hospital > 20 h
Cr	No distortion of the inner membrane ^a^	Limb ischemia	Lac > 1.9 mmol/L
FIB	Number of breaks	Ischemic manifestation in abdominal vasculature	WBC > 14.2 × 10^9^/L
D-Dimer	Number of branch vessels involved	Limb blood pressure ^e^	AST > 80 U/L ^c^
AST	Difference in blood pressure of extremities > 20 mmHg	Aortic branch vessels involved ^f^	Type A interlayer classification
	True cavity area ^d^		Creatine kinase value

^a^ The inner diaphragm rotated clockwise or counterclockwise ≤ 90°. ^b^ The specified quantity data were converted into binary variables according to the optimal cut-off value of its ROC curve. ^c^ Liver damage was determined according to the normal high limit of more than 2 times transaminase (80 U/L) in the actual clinical work. We converted the AST into a binary classification variable. ^d^ The relevant data were measured on the widest cross-section of the ascending aorta. ^e^ Including systolic and diastolic blood pressure in the extremities. ^f^ It contains all the branches of the aorta.

**Table 2:** Multivariate logistic regression analysis of risk factors for dissection rupture within 72 h after CTA.

**Risk Factor**	**Regression Coefficient (β)**	**Waldx^2^**	***p***	**OR** **Value**	**95% CI**
Age > 63 years	1.687	8.487	0.004	5.403	1.737–16.810
Women	1.769	10.131	0.001	5.865	1.973–17.432
Ventilator-assisted ventilation	3.052	14.203	0.010	21.156	4.326–4.326
AST value > 80 U/L	1.594	5.156	0.023	4.926	1.244–19.506
No distortion of the inner membrane	1.571	9.685	0.002	4.811	1.789–12.940
Aortic sinus diameter > 41 mm	0.927	3.790	0.052	2.527	0.994–6.426
Widest diameter > 48 mm	1.320	8.751	0.003	3.745	1.561–8.982
Ratio of false lumen area to true lumen area > 2.12	1.935	13.336	0.010	6.927	2.451–19.574
Lac value > 1.9 mmol/L	2.281	20.955	0.010	9.782	3.684–25.973
WBC value > 14.2 × 10^9^/L	1.225	7.672	0.006	3.404	1.431–8.101

**Table 3:** Performance comparison of each model. (Estimates and 95% confidence intervals.).

Model Name	AUC	Accuracy	Precision	F1-Score	Specificity	Recall
LR	0.91 (0.90–0.94)	0.85 (0.84–0.85)	0.90 (0.86–0.93)	0.88 (0.87–0.91)	0.86 (0.85–0.88)	0.90 (0.89–0.91)
RF	0.94 (0.90–0.97)	0.90 (0.85–0.93)	0.92 (0.90–0.97)	0.89 (0.86–0.90)	0.91 (0.90–0.93)	0.95 (0.90–0.98)
SVM	0.89 (0.86–0.94)	0.83 (0.82–0.85)	0.78 (0.76–0.79)	0.77 (0.73–0.78)	0.85 (0.81–0.85)	0.88 (0.83–0.91)
CNN	0.99 (0.95–0.99)	0.90 (0.88–0.91)	0.90 (0.89–0.92)	0.90 (0.89–0.93)	0.90 (0.87–0.93)	0.90 (0.88–0.92)

Abbreviation: SVM support vector machine, LR logistic regression, RF random forest, CNN convolutional neural network.

## Data Availability

The datasets used and/or analyzed during the current study are available from the corresponding author on reasonable request. It can also be downloaded via the link below (https://github.com/xiangxiangzhuyi/Prediction-of-Acute-Aortic-Dissection-Rupture (accessed on 16 December 2022)).
